# Enhancing content and language integrated learning in post-secondary vocational education

**DOI:** 10.1186/2193-1801-4-S2-O3

**Published:** 2015-11-27

**Authors:** Angel Garralda Ortega, Janet Man-wai Cheung, Michelle Yuen-shan Fong

**Affiliations:** School of General Education and Languages, Technological and Higher Education Institute of Hong Kong, Hong Kong; Research Support Unit, Vocational Training Council, Hong Kong

## Background

As the Final Report of the Language Education Review highlights, students' low level of English proficiency seriously affects learning in Hong Kong's tertiary institutions and hinders students' potential to fully benefit from English-medium education. This problem is especially serious in self-funded degree programmes, as the students admitted onto these programmes very rarely have scores above 3 on the DSE examinations. This paper reports on a pilot study conducted at THEi aimed at combining Content and Language Integrated Learning (CLIL) and IT innovation to support cognitive and linguistic scaffolding through blended learning.

## Methods

The potential benefits of this pedagogical innovation are illustrated in the context of a Year 1 General Education Core Module, *The Human Spirit*. We investigated learner engagement and the attitudes of teachers and students towards CLIL and blended learning, based on participant observation, Web analytics and survey data [1]. Lectures and tutorials on the human spirit were observed before and during the implementation of CLIL through blended learning, and tutor and student views regarding delivery modes and learning styles were collected during informal discussions throughout the course [2]. Student presentations given in the tutorials, which followed the blended learning component, were also recorded for further study [3]. Google Analytics was used to assess user engagement in e-learning both quantitatively and qualitatively, as the students' work was stored in the system [4]. Forty students selected from among those who were most actively engaged in blended learning were surveyed to explore their perceptions of whether blended learning was more time consuming and more effective than traditional lectures and tutorials.

## Results

As the teachers' perceptions of key components of blended learning, such as flipping classes, proved to be ambivalent during the course of the project, blended learning was eventually made optional alongside the traditional delivery mode of lectures and tutorials. Student engagement in blended learning varied from group to group, as reflected in the number of page views and the activities engaged in. Nearly 60% of the students used some form of blended learning and almost 40% tried both the reading and the listening component. The listening component proved to be slightly more popular and was also considered more useful than the reading component, as reflected in the level of user engagement and in a perception survey conducted at the end of the project.

More than half of the students expressed that blended learning improved their comprehension of the subject, helped them to develop language proficiency and eased their workload. However, when asked to choose between blended learning and traditional learning, only about half of the respondents preferred this novel delivery mode and over a third remained non-committal.

## Conclusions

The results of this pilot project indicate that using blended learning in combination with CLIL may be effective, but factors such as pedagogical constraints, learner motivation and teacher/student attitudes and agendas must be carefully considered for pedagogical innovation to succeed.

## References

[1] Cummins J, Alatis JE, Staczek JJ (1985). The construct of language proficiency in bilingual education. Perspectives on Bilingualism and Bilingual Education.

[2] Rogers EM (2003). Diffusion of Innovations.

[3] Standing Committee on Language Education and Research (SCOLAR) (2003). Action plan to raise language standards in Hong Kong: final report of language education review. Hong Kong: Hong Kong Government Documents.

